# Clinical research updates

**DOI:** 10.1111/camh.70014

**Published:** 2025-07-11

**Authors:** Marinos Kyriakopoulos, Maria Papadaki, Aikaterini Zeza, Niki‐Stavroula Panagiotopoulou

**Affiliations:** ^1^ 1st Department of Psychiatry National and Kapodistrian University of Athens Athens Greece; ^2^ Department of Child and Adolescent Psychiatry Institute of Psychiatry Psychology and Neuroscience, King's College London London UK; ^3^ South London and Maudsley NHS Foundation Trust London UK

## Can a warm and supportive adult protect against mental health problems among children with experience of adversity?

Maria Papadaki

Understanding why some children exposed to adverse childhood experiences (ACEs) develop mental health difficulties while others remain resilient is important for designing effective preventive strategies. It has been suggested that the presence of a warm and supportive adult may protect against mental health difficulties in such cases by moderating the negative effects of early adversity. However, it remains unclear whether such an association is causal or whether it may be accounted for by genetic or environmental factors.

Stock et al. (2025) investigated this question using data from the Environmental Risk (E‐Risk) Longitudinal Twin Study, a representative UK‐based birth cohort of 2232 same‐sex twins. ACEs were assessed prospectively covering the period from age 5 to age 12 years. Maternal warmth was evaluated at two timepoints, at ages 5 and 10 years, using structured speech samples from mothers. Adult support in children's lives was gathered through self‐reports at age 12. Mental health outcomes were assessed at age 12 through interviews with parents and teachers and at 18 years through self‐reports from the participants using p‐factor as a standardized index.

Initial phenotypic analyses revealed that, among children exposed to ACEs, those who experienced greater maternal warmth and adult support had lower levels of emotional and behavioral problems in early adolescence, as well as lower p‐factor scores at 18 years. However, when a monozygotic twin‐difference analysis was applied, the strength of these associations was significantly reduced: by about 70% for maternal warmth and 81% for adult support. In twin pairs equally exposed to ACEs, the twin who experienced more maternal warmth and support from adults tended to have similar mental health outcomes to their co‐twin.

The authors identified some limitations to their investigation, including the measurement of adult support through children's self‐report, which may be affected by genetically influenced perception bias, the use of difference scores in the twin differences design, which may induce error in the measurement of protective factors, and the possible non‐generalisability to singletons.

Nevertheless, this study suggests that the observed effects of the support by a warm and supportive adult in children experiencing ACEs are largely due to genetic and environmental confounding rather than independent causal effects, which highlights the need for multifaceted interventions. Efforts to improve children's mental health after adversity should not focus exclusively on strengthening adult–child relationships but should also address broader family risk factors and inherited vulnerabilities.

## Reference

Stock, S.E., Lacey, R.E., Arseneault, L., Caspi, A., Crush, E., Danese, A., & Baldwin, J.R. (2025). Can a warm and supportive adult protect against mental health problems amongst children with experience of adversity? A twin‐differences study. *Journal of Child Psychology and Psychiatry*, 66, 650–658. doi: 10.1111/jcpp.14070.

## Is asking preadolescents about suicide associated with increased suicidal thoughts?

Aikaterini Zeza

The phenomenon of increasing rates of suicidal ideation and behaviors (STBs) among preadolescents has raised concerns about potential triggers. It is conceivable that repeated suicide‐risk screening has adverse iatrogenic effects and may increase the risk of such thoughts among children, especially those who had not previously shown any suicidal behavior.

Hennefield at al. (2025) aimed to explore this possibility within the Pediatric Suicidality Study (PED‐SI), which includes children participating in earlier research on preschool‐onset depression. A total of 192 children aged 8–12 years were divided into a lower‐risk group (*n* = 68) with no prior suicidal thoughts or behaviors and a higher‐risk group (*n* = 124) with history of suicidal ideation or self‐harm. Participants completed a modified version of the Ask Suicide‐Screening Questions (ASQ) on suicidal thoughts in the past week via e‐mail or text. The frequency of completion was monthly for the lower‐risk group and weekly for the higher‐risk group.

The study found that 1.6% of responses were positive for suicidal thoughts in the lower‐risk group and 7% of responses were positive in the higher‐risk group. There was no evidence that the number of completed screenings increased the likelihood of positive responses in either group. Screening did not seem to cause an increase of suicidal thoughts nor predict a greater chance of endorsing suicidal ideation at the next timepoint. Notably, completion rates were high, suggesting that digital tools for mental health monitoring are feasible and acceptable for this age group. Moreover, screening did not cause distress or withdrawal from participation. In fact, children who endorsed suicidal thoughts were sometimes more likely to complete future surveys, indicating a potential openness to mental health communication when it is relevant to their experience. These results support calls for broader routine suicide‐risk screening in pediatric care settings which may allow for timely intervention.

The authors identified several limitations including the sample being predominantly White, the study not assessing suicide attempts, and the highest frequency of the screening being weekly. Furthermore, because there was no control group, it was not possible to evaluate whether screening lowered or raised the risk of suicidal thoughts. Still, the low rate of new suicidal ideation among low‐risk children, and lack of escalation in high‐risk participants, strongly suggests safety and potential benefit.

This study adds to the body of evidence suggesting that suicide‐risk screening is safe in preadolescents, even when conducted regularly over an extended period. The findings support the expansion of screening programs and the development of digital tools for monitoring youth mental health, particularly for high‐risk children who may benefit from early and sustained attention.

## Reference

Hennefield, L., Luking, K.R., Tillman, R., Barch, D.M., Luby, J.L., Thompson, R.J. (2025). Asking preadolescents about suicide is not associated with increased suicidal thoughts. *Journal of the American Academy of Child and Adolescent Psychiatry*: S0890‐8567(25)00178‐9. doi: 10.1016/j.jaac.2025.03.025.

## Antipsychotic medication in pregnancy and neurodevelopmental outcomes in children

Niki‐Stavroula Panagiotopoulou

Antipsychotic medications are widely prescribed, including during pregnancy, raising concerns about potential long‐term effects on offspring. Although these drugs are not considered teratogenic, their impact on child neurodevelopment remains uncertain. Research in this area is limited by the lack of clinical trials involving pregnant women and the difficulty of disentangling medication effects from those of maternal mental illness and other confounding factors.

Kaplan et al. (2025) conducted a systematic review to assess whether prenatal exposure to antipsychotic (AP) medications is associated with adverse neurodevelopmental outcomes in children. They identified 16 studies, six cohort and ten register‐based, published through September 2024. These studies examined motor, cognitive, behavioral, psychiatric, and academic outcomes. Study quality was evaluated using the Newcastle‐Ottawa Scale, with a mean score of 7.1 out of 9.

The review found that, after adjusting for key confounders, most studies did not report significant neurodevelopmental differences between AP‐exposed and unexposed children. Large population‐based studies showed no increased risk for ADHD, autism spectrum disorder, or other neurodevelopmental disorders. Smaller cohort studies similarly found no associations with cognitive or psychological outcomes. Early motor or behavioral delays were more commonly reported in studies with short follow‐up periods, but these effects did not persist in longer‐term studies. This suggests that such early deficits may be transient and possibly related to neonatal withdrawal or temporary neurodevelopmental effects following discontinuation of AP exposure at birth. However, the influence of maternal psychiatric illness on outcomes remains a key limitation, particularly in studies that lacked appropriate control groups.

Interpreting these findings requires careful consideration of confounding variables. Children of mothers with severe mental illness already face increased risks for developmental challenges that may overlap with outcomes attributed to AP exposure. Studies that included psychiatric control groups tended to report only transient motor delays in infancy, with no long‐term differences in academic performance or neurodevelopment. Other confounders – such as smoking, substance use, socioeconomic status, and maternal education – were inconsistently addressed across studies. In several cases, associations present in unadjusted analyses disappeared after full statistical adjustment.

Importantly, pharmacological treatment of maternal mental illness may also provide protective effects. One study found a reduced risk of ADHD in children whose mothers continued AP treatment during pregnancy, compared to those who discontinued. Although research on protective effects of antipsychotics is limited, similar findings from antidepressant studies suggest that effective maternal treatment may mitigate developmental risks. This highlights the importance of considering both potential harms, including metabolic syndrome and gestational diabetes, and potential benefits to the mother and the child, when evaluating the use of APs in pregnancy. Clinical decisions should be individualized and based on shared risk–benefit discussions.

## Reference

Kaplan CA, Poels EMP, van den Heuvel MI, Bijma HH, Bergink V, Rommel A‐S, Robakis T, Systematic Review: Antipsychotic Medication in Pregnancy and Neurodevelopmental Outcomes in Children, *Journal of the American Academy of Child & Adolescent Psychiatry* (2025), doi: 10.1016/j.jaac.2025.04.008.

## Conflict of interest statement

M.K. is the *CAMH* Associate Editor for Clinical Research Updates. The editor thanks the contributors for this issue's Clinical Research Updates. The editor has declared that he has no competing or potential conflicts of interest.

## Ethics statement

No ethical approval was required for these updates.

## Data Availability

Data sharing is not applicable to this article as no new data were created or analyzed.

